# Molecular characterization of doubled haploid lines derived from different cycles of the Iowa Stiff Stalk Synthetic (BSSS) maize population

**DOI:** 10.3389/fpls.2023.1226072

**Published:** 2023-07-27

**Authors:** Alejandro Ledesma, Fernando Augusto Sales Ribeiro, Alison Uberti, Jode Edwards, Sarah Hearne, Ursula Frei, Thomas Lübberstedt

**Affiliations:** ^1^Department of Agronomy, Iowa State University, Ames, IA, United States; ^2^USDA-ARS, Corn Insects and Crop Genetics Research Unit, Ames, IA, United States; ^3^International Maize and Wheat Improvement Center (CIMMYT), El Batan, Texcoco, Mexico

**Keywords:** zea mays L., diversity, genetic resources, homozygous lines, genetic diversity

## Abstract

Molecular characterization of a given set of maize germplasm could be useful for understanding the use of the assembled germplasm for further improvement in a breeding program, such as analyzing genetic diversity, selecting a parental line, assigning heterotic groups, creating a core set of germplasm and/or performing association analysis for traits of interest. In this study, we used single nucleotide polymorphism (SNP) markers to assess the genetic variability in a set of doubled haploid (DH) lines derived from the unselected Iowa Stiff Stalk Synthetic (BSSS) maize population, denoted as C0 (BSSS(R)C0), the seventeenth cycle of reciprocal recurrent selection in BSSS (BSSS(R)C17), denoted as C17 and the cross between BSSS(R)C0 and BSSS(R)C17 denoted as C0/C17. With the aim to explore if we have potentially lost diversity from C0 to C17 derived DH lines and observe whether useful genetic variation in C0 was left behind during the selection process since C0 could be a reservoir of genetic diversity that could be untapped using DH technology. Additionally, we quantify the contribution of the BSSS progenitors in each set of DH lines. The molecular characterization analysis confirmed the apparent separation and the loss of genetic variability from C0 to C17 through the recurrent selection process. Which was observed by the degree of differentiation between the C0_DHL versus C17_DHL groups by Wright’s F-statistics (FST). Similarly for the population structure based on principal component analysis (PCA) revealed a clear separation among groups of DH lines. Some of the progenitors had a higher genetic contribution in C0 compared with C0/C17 and C17 derived DH lines. Although genetic drift can explain most of the genetic structure genome-wide, phenotypic data provide evidence that selection has altered favorable allele frequencies in the BSSS maize population through the reciprocal recurrent selection program.

## Introduction

The maize Iowa Stiff Stalk Synthetic (BSSS) population has undergone recurrent selection since 1939. This population was developed by intermating 16 inbred lines selected for superior stalk quality (Sprague and Jenkins, 1943). The C0 base population was subjected to multiple cycles of recurrent selection. Currently, C19 is available. The BSSS maize population has been under recurrent selection for increased grain yield, low grain moisture at harvest and increased resistance to root and stalk lodging. Phenotypic and genotypic changes have been observed in this population (Messmer et al., 1991; Labate et al., 1999; Hagdorn et al., 2003; Edwards, 2011; Gerke et al., 2015), suggesting loss of genetic variability from C0 to more advanced cycles of selection. As noted by Ledesma (2020) when they evaluated the level of phenotypic diversity and identified significant SNPs by GWAS in different cycles of recurrent selection of the BSSS population based on doubled haploid (DH) lines. Alleles present in a heterogeneous population of heterozygous individuals can be fixed in homozygous and homogenous DH lines and part of the genetic diversity in a population can be harnessed for breeding by production of DH lines (Böhm et al., 2017). However, the success of this approach relies on the choice of promising populations and extensive characterization of the produced DH lines (Böhm et al., 2017). The combination of DH technology with high-throughput genotyping drives progress in major maize breeding programs today (Andorf et al., 2019) and has been applied in this study to understand the evolution and genotypic composition of different cycles of BSSS maize population.

Molecular markers like single nucleotide polymorphisms (SNPs) have proven to be valuable for the characterization of maize germplasm and their application becoming more feasible over the past two decades due to the availability of new, high density and affordable genotyping technologies (Lu et al., 2009). Useful measures of the quality of genetic markers’ polymorphisms are the expected heterozygosity (H_exp_). Expected heterozygosity (H_e_) is defined as the probability that any two alleles at a single locus, chosen randomly from the population, are different from each other (Nei and Roychoudhury, 1974; Nei, 1978).

The genetic relationship based on genetic distance was first defined by Nei (1973) as the difference between two samples that can be described by allelic variation, meaning that genotypes with many similar genes have a smaller genetic distance between them. The degree of genetic differentiation using the fixation index (F_ST_) is a standard measure for the degree of genetic differentiation among subpopulations (Wright, 1951). The F_ST_ provides important insights into the evolutionary processes that influence the structure of genetic variation within and among populations (Holsinger and Weir, 2009). The F_ST_ estimates can identify regions of the genome that have been targeted for selection (Beckett et al., 2017; Guo et al., 2021; Wijayasekara and Ali, 2021). The comparison of F_ST_ from different genome regions can provide insights into populations demographic history (Holsinger and Weir, 2009).

Characterizing and understanding the genetic diversity and relationships of lines within a breeding program is essential for germplasm improvement (Andorf et al., 2019). Molecular markers have been used to estimate the relative strengths of evolutionary forces: mutation, natural selection, migration and genetic drift (Ouborg et al., 1999) and a possible loss of genetic diversity in specific populations, including BSSS (Gerke et al., 2015). Gerke et al. (2015), when evaluating different cycles of selection in a recurrent selection program, found that the populations steadily decreased in genetic diversity within populations and increased in genetic differentiation between populations mainly due to genetic drift and selection. According to the same authors, the C0 population has drifted away from the BSSS founders, despite the absence of intentional selection during the creation and maintenance of C0. In our study, we used different methods proposed as genetic diversity and differentiation measures using genotypic information. Additionally, we used developed DH lines instead of individual heterozygous plants representing the different cycles of the BSSS population.

Population structure is referred to as any form of relatedness among subgroups within the overall sample, including ancestry differences or cryptic relatedness (Sul et al., 2018). Population structure analysis involves grouping of individuals into subpopulations based on shared genetic variants and can be assessed through principal component analysis (PCA). PCA can identify differences in ancestry among populations and individuals, regardless of the historical patterns underlying population structure (Price et al., 2006; Zhu and Yu, 2009), since PCA clusters individuals based on the number of markers that are identical by state among them. Based on this grouping and relationship information among individuals, plant breeders can direct crosses, avoiding the mating closely related individuals and providing a reduction in inbreeding in their breeding programs. For instance, kinship coefficients have been used to estimate the genetic relationships within populations and to estimate the genetic contribution of a set of parents to its descendants (Yang et al., 2011; Ertiro et al., 2017; Wegary et al., 2019). Therefore, the estimation of kinship coefficients represents a way to utilize breeding resources more efficiently (Beckett et al., 2017).

An identity by descent (IBD) segment refers to DNA segments descended from common ancestors and could be useful to estimate the genetic relationships in a population. IBD occurs when identical alleles are inherited from a common ancestor and constitutes a measure of the degree of relationship between individuals (Wright, 1922). The estimation of the degree of the relationship depends on the description of an ancestral population, which by definition, is assumed to be the base from where past ancestry is no longer accounted (Wright, 1922). With the advent of high-throughput genotyping technologies, IBD segments can be estimated at a molecular scale. The identification of shared segments in the genome and haplotype information has been used for a range of purposes, including the quantification of inbreeding (Keller et al., 2011), identification of patterns of inheritance (Kirin et al., 2010), genotype imputation and haplotype inference (Browning and Browning, 2007), genetic characterization and diversity analysis (Nelson et al., 2008), the genetic contribution of a set of founder lines in commercial maize breeding programs (Coffman et al., 2019), and to improve the accuracy of genome-wide association analysis (GWAS; Maldonado et al., 2019) and genomic prediction (Won et al., 2020).

In this study of the BSSS, we propose to determine how much of the genomic variation in C0 has been lost during the selection process. C0 may be a reservoir of untapped favorable genetic diversity for previously unselected traits. Developing DH lines from earlier cycles of selection could be an alternative approach to conventional breeding for introduction of diversity into related elite lines. Genetic heterogeneity and high genetic load present in C0 could be overcome by production of DH lines (Böhm et al., 2017) to unlock genetic diversity. Diversity may have been lost not only due to selection can also be attributed to genetic drift or genetic hitchhiking effects, since no new genetic material was intentionally introduced into the BSSS population.

Our overall question in this and a companion paper Ledesma (2020) was whether potentially useful genetic diversity is available in earlier cycles of selection in the recurrent selection process, which may be more accessible sources of alleles compared with founding non-adapted landraces and other such genetic resources. Here, we used SNP markers to i) estimate and compare the genetic diversity within different subsets of DH lines derived from the BSSS maize population after different cycles of selection, ii) determine, if genetic diversity was lost from C0 to C17, iii) assess the genetic relationships and genetic divergence within and among the cycles of selection, and iv) perform a haplotype analysis based on IBD segments to quantify the contribution of the progenitors to each set of DH lines.

## Materials and methods

### Breeding populations

Three synthetic populations BSSS, BSSS(R)C17, and BSSS/BSSS(R)C17 representing different cycles of selection in the reciprocal recurrent selection program with BSSS, and the Iowa Corn Borer Synthetic number 1 (BSCB1) were used to develop DH lines. The synthetic BSSS corresponds to the unselected base population (C0) formed by intermating 16 inbred lines selected for above average stalk quality in 1934 (Sprague, 1946). The C0 seed used came from subsequent cycles of seed multiplication in C0 for maintenance over time. The BSSS(R)C17 (C17) population corresponds to the seventeenth cycle of reciprocal recurrent selection with BSCB1 (Penny and Eberhart, 1971; Lamkey, 1992; Keeratinijakal and Lamkey, 1993; Edwards, 2011). Finally, BSSS/BSSS(R)17 was created by crossing plants from BSSS with plants in BSSS(R)C17 and intermating to create the BSSS/BSSS(R)C17 population (C0/C17). We also included in this study 14 (A3G-3-3-1-3, CI 540, I-159, IL12E, Oh 3167B, Os 420, Tr 9-1-1-6, WD 456, I224, LE 23, 461, Hy, AH83, CI 187-2) of the 16 known progenitors of the BSSS, plus the two parents (Fe and B2) of the F1B1 line. That is, a total of 16 progenitors were included in the study. Seed from the progenitor lines CI 617 and F1B1 were not available.

### DH line development

Randomly selected individuals within each population were pollinated with a maternal haploid inducer BHI301 (Almeida et al., 2020) in an isolation field to generate the haploid seed. Seed produced from these plants was screened and kernels expressing the *R*-*nj* marker gene in the endosperm, but not in the embryo, were classified as haploid kernels. The haploid seed was germinated in plug trays in the Department of Agronomy greenhouse. Once seedlings developed 2-3 leaves, a colchicine treatment was applied following the protocol used by the DH Facility at ISU (Vanous et al., 2017). Two days after the colchicine treatment, haploid seedlings were transplanted in the field at the Agricultural Engineering and Agronomy Research Farm, Boone, IA. At flowering stage, putative DH_0_ plants shedding pollen were self-pollinated to produce DH_1_ seed. Seed multiplication was performed during subsequent generations and lines were screened for uniformity and discarded if they were segregating or variable. In total, 132 DH lines from BSSS(R)C0 (C0_DHL), 185 DH lines from BSSS(R)17 (C17_ DHL), and 170 DH lines from BSSS(R)C0/BSSS(R)17 (C0/C17_DHL) were obtained. The DH lines were developed by the DH Facility at ISU (http://www.plantbreeding.iastate.edu/DHF/DHF.htm ).

### Genotyping and quality control

Genomic DNA was extracted from DH line seedlings established in a greenhouse. Leaf tissue samples from three plants per DH line were collected at the 3-4 leaf developmental stage, and DNA extraction was done using the standard International Maize and Wheat Improvement Center (CIMMYT) laboratory protocol (Warburton, 2005). Genotyping was carried out using the Diversity Arrays Technology sequencing (DArT-seq) method (Kilian et al., 2012) provided by the Genetic Analysis Service for Agriculture (SAGA) laboratory at CIMMYT. DArT-seq is a high-throughput, robust, reproducible, and cost-effective genotyping technology based on genome complexity reduction using a combination of tailored restriction enzymes, followed by multiplexed sequencing of resulting libraries to simultaneously assay thousands of markers across the genome (Sansaloni et al., 2011). Across the samples assessed a total of 51,418 SNP markers were generated, of these 32,929 SNP markers were successfully aligned to the B73 RefGen_v4 (Jiao et al., 2017). Monomorphic and multi-allelic markers were removed. Un-imputed data without filtering for minor allele frequency (MAF) were used for further analyses.

The inbred line B73 was used as technical control and was repeated in seven separate plates to verify assay reproducibility. The resulting SNP core set was 24,885 SNP markers corresponding to 487 DH lines (132 C0_DHLs, 170 C0/C17_DHLs, 185 C17_DHLs) and 15 progenitors). The progenitor CI 187-2 was omitted because of heterozygosity greater than 8.8% (not expected in inbred lines) and was removed from further analyses. After this point, only 15 progenitors with low heterozygosity were used in the study.

### Genotypic data analysis

Minor allele frequency analysis for each locus across the genotypes was calculated using the 24,885 SNP markers with the function ‘Geno summary’ analysis tool in the software TASSEL v.5.2.64 (Bradbury et al., 2007). The expected heterozygosity (H_exp_) was calculated to quantify the genetic variation in the maize lines sampled. The expected heterozygosity is defined as the probability that two alleles randomly chosen from the test sample are different (Nei, 1978). The expected heterozygosity was calculated using the R package “Poppr” (Kamvar et al., 2014), with the following formula: $H_{exp}=\left(\frac{n}{n-1}\right)1-\sum_{i=1}^{k}p_{i}^{2}$ , where *p* is the allele frequency at a given locus, which goes from *i* to *k*, and *n* is the number of observed alleles for each locus (Nei, 1978).

The computation of dissimilarity coefficients or Euclidean genetic distance (Gower and Legendre, 1986) between DH lines and progenitor groups was performed with the 24,885 SNP markers using the R package “Poppr” (Kamvar et al., 2014). The genetic distances were calculated based on the average genetic distance of all lines within each other group. Cluster analyses were performed to subdivide the three sets of DH lines and the progenitor group into genetic subgroups using the Unweighted Pair Group Method with Arithmetic mean (UPGMA). Finally, dendrograms were constructed based on genetic distances using the visualization software Interactive Tree of Life (iTOL; Letunic and Bork, 2019).

To assess the degree of genetic differentiation between the groups of DH lines and the progenitors, we used the Wright’s F-statistics (F_ST_) on a per locus basis using the methodology described by Weir and Cockerham (1984), which accounts for unequal population sizes and sampling variances since heterozygous loci are weighted by the number of alleles observed in each population. The R package “hierfstat” (Goudet, 2005) was used to obtain estimates of F_ST_. The F_ST_ values can range from zero to one, where high F_ST_ values showed a considerable difference in the allele frequency among two populations.

The pairwise relative kinship for all 487 DH lines and the 15 progenitors was estimated based on the 24,885 SNP markers using the software TASSEL v.5.2.64 (Bradbury et al., 2007) using the centered_IBS method (Endelman and Jannink, 2012). The relative kinship reflects the approximate degree of identity between two given individuals over the average probability of identity between two random individuals (Yu et al., 2006). The pairwise relative kinship was used to measure the genetic resemblance among individuals. A relative kinship close to zero indicates no relationship, and values close to one indicate a close relationship. Marker-based kinship coefficients show the relationship among lines based on genotypic information and rely on the marker allele frequencies in the reference population, which in practice is not known (Wang, 2014). However, 15 of the 16 progenitors of BSSS are known. These estimates commonly use the sample of genotyped individuals as the reference population, resulting in estimates that two homologous genes within or between individuals are shared by descent (Wang, 2014). Marker-based estimation of kinship coefficients can result in negative values. Wang (2014) states that the kinship coefficient’s negative values could be interpreted as a lower probability that two homologous alleles are shared by descent compared with the probability that two alleles are taken at random from the reference population.

The 487 DH lines and the 15 progenitors were known to belong to the four subpopulations BSSS(R)C0, BSSS(R)C17, BSSS(R)C0/C17 and the progenitor groups, respectively. To examine the overall population structure across all lines, we performed a principal component analysis (PCA). PCA analysis allows the classification of individuals into genetically similar groups. PCA relies on reducing dimensionality by using principal components to maximize genetic variability (Price et al., 2006). Each principal component will account for a percentage of the total genetic variance by grouping the individuals into clusters with similar genetic information. After reducing dimensionality, a linear regression model was fitted to each of the axes of variation, and the residuals were extracted to compute associations (Price et al., 2006). PCA avoids any prior information about individual ancestries, the population of origin, and assumptions about the data, handling genome-wide data for thousands of individuals (Paschou et al., 2007). PCA was performed using the software GAPIT v.3 (Lipka et al., 2012). Bayesian Information Criterion (BIC; Schwarz, 1978) was used to identify the optimal number of principal components by selecting the lowest BIC model. The principal component results were used to display the first two principal components in R software (R Core Team, 2021).

The average linkage disequilibrium (LD) decay among SNP markers for each chromosome was determined in each group of DH lines using the squared Pearson correlation coefficient (r^2^) among alleles at two loci, for all possible combinations of alleles, and then weighting them according to the allele frequency. P-values were determined by a two-sided Fishers Exact test (Bradbury et al., 2007). The option “Full Matrix LD” on TASSEL v.5.2.64 was used to calculate LD for every combination of sites in the alignment (Bradbury et al., 2007). The resulting data were imported into R (R Core Team, 2021) to create LD decay plots and fit a smooth line using Hill and Weir expectations of r^2^ among adjacent sites (Hill and Weir, 1988).

To quantify the progenitors genetic contributions to the different sets of DH lines, we used high-resolution detection of identity by descend (IBD) segments. An IBD segment refers to DNA segments descended from common ancestors. IBD occurs when identical alleles are inherited from a common ancestor and could be used to estimate the genetic contribution. Estimation of IBD segments with genotypic data allows the quantification of the proportion of the covered genome descended from each progenitor. For the genetic contribution and the average LD decay among SNP marker analysis, a different filtering process of the genotypic data was conducted to have the most reliable SNP markers and ensure genotype concordance. From the 32,929 SNP markers successfully called within the B73 RefGen_v4 (Jiao et al., 2017). SNP markers with missing information rate above 10%, duplicated and monomorphic markers were removed in TASSEL v.5.2.64 (Bradbury et al., 2007). Genotypes were phased and imputed by using Beagle v.5.1 (Browning et al., 2018). Physical distance for each marker was converted to genetic distance using a dense 0.2 cM resolution map (Ogut et al., 2015), with on average 1385.6 kb per cM. After completing filtering and quality control, the genotypic data file contained 10,344 SNP markers for each of the 502 genotypes (487 DH lines and 15 progenitors) covering 2102.7 Mb (1,517.5 cM) of the genome and with one marker per 203.2 kb on average. The SNP markers not included in an IBD segment were referred to as non-IBD markers, while those within the IBD segment were labeled with the progenitor sharing the segment. The proportion of the genome descended from a progenitor was calculated by dividing the total number of SNP markers classified as IBD by the total number of polymorphic SNP markers. Regions in the genome (IBD segments) that have been inherited from the progenitor were identified with the identity by descent linkage disequilibrium (IBDLD) program v.3.38 (Han and Abney, 2011; Han and Abney, 2013). The IBDLD program uses a probabilistic approach with a hidden Markov model to estimate IBD segments in pairs of individuals. The IBDLD program further expresses the emission probability conditioned on the true genotype of *n* previous loci to account for linkage disequilibrium (Han and Abney, 2011). IBD segments were constrained for each pair of individuals to have a minimum length of 350 kb, have more than 10 SNP markers and SNP markers with an IBD probability above 70%. These parameters force the segment to be a long IBD segment, avoiding segments formed by an occasional genotyping error or missing genotype occurring in otherwise-unbroken segments that could underestimate IBD segments for each pair of individuals (McQuillan et al., 2008).

## Results

The initial number of SNP markers in the DArT-seq data set was 51,418. A total of 32,929 SNP markers were successfully called within the B73 RefGen_v4 (Jiao et al., 2017). After removing monomorphic and multi-allelic markers, the final SNP marker data set included 24,885 SNPs distributed across the ten chromosomes. The SNP density varied among chromosomes ranged from 3,976 to 1,688 markers on chromosome 1 and 10, respectively (**Table 1**). Heterozygosity varied from 1.2% on chromosomes 2 and 7 to 1.6% on chromosome 9, with a mean value of 1.3% across the ten chromosomes. We found heterozygous loci among the DHLs which ranged from 0.40 (C17_DHL045) to 2.24% (C0/C17_DHL146; **Supplemental Table S1**).

**Table 1:** Genotypic data summary for the 24,885 SNP markers and the entire panel of DH lines derived from different BSSS selection cycles.

Chromosome number	Number of SNP markers	Heterozygosity rate (%)
1	3,976	1.3
2	2,978	1.3
3	2,721	1.2
4	2,453	1.5
5	2,798	1.3
6	1,929	1.4
7	2,203	1.2
8	2,105	1.5
9	2,034	1.6
10	1,688	1.4
Genome-wide	24,885	1.3

### Molecular characterization analysis

**Figure 1:**
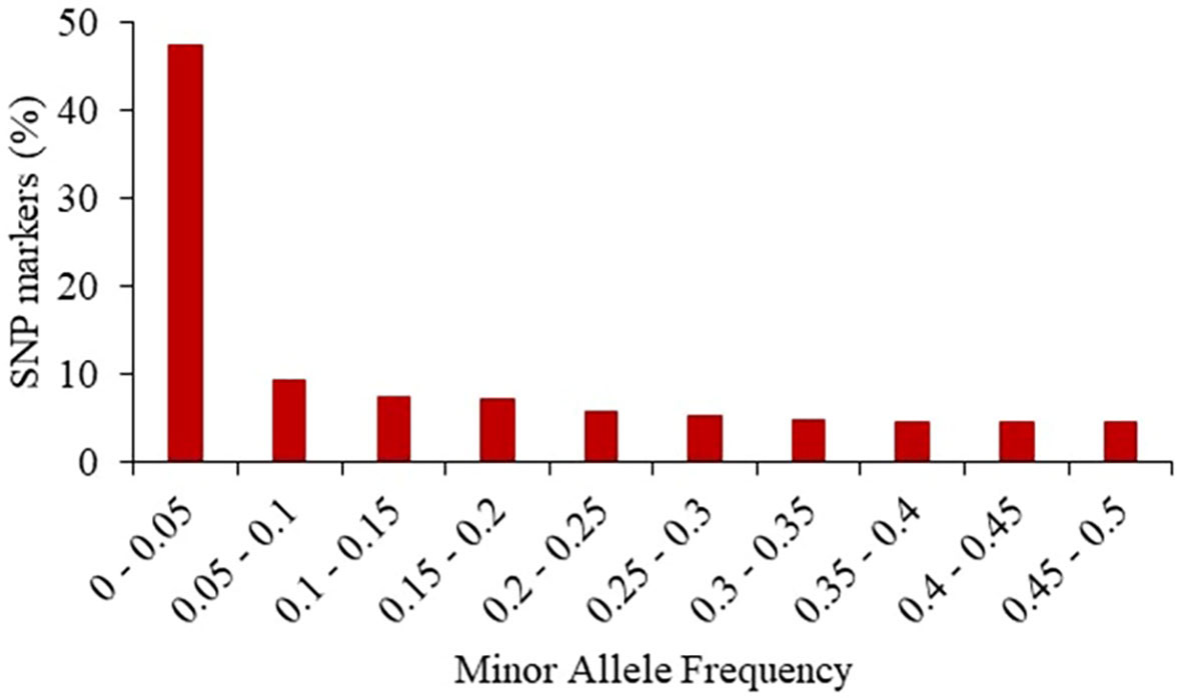
Frequency distribution of minor alleles in the entire panel of 487 BSSS DH lines and the 15 progenitors based on 24,885 SNP markers.

The 24,885 SNP markers were polymorphic with a MAF greater than zero (**Figure 1**). The average MAF was 0.19, 0.16, 0.13 and 0.07 in the progenitor, C0_DHL, C0/C17_DHL and C17_DHL groups, respectively (**Table 2**). The highest expected heterozygosity was in the progenitor’s group (H_exp_ = 0.28), followed by the C0_DHL group with H_exp_ = 0.21 (**Table 2**). The lowest expected heterozygosity value was observed in the C17_DHL group as expected. In comparison, the group C0/C17_DHL had an expected heterozygosity value of H_exp_ = 0.19. The MAF and expected heterozygosity values all ranked populations in the same order. Higher values in progenitor and C0_DHL group were expected, which represents higher allelic variation in relation to the C0/C17_DHLs and C17_DHL groups. These values in the C0/C17_DHL group (F1 cross) were according with the expectation and were predictable values since we knew the parent populations (C0_DHL and C17_DHL) values.

**Table 2:** Average Minor Allele Frequency (MAF) and expected heterozygosity (H_exp_) within each group of DH lines and progenitors.

Group	Genotypes	Average MAF	H_exp_
**Progenitors**	15	0.19 ± 0.001	0.28 ± 0.001
**C0_DHL**	132	0.16 ± 0.001	0.21 ± 0.001
**C0/C17_DHL**	170	0.13 ± 0.001	0.19 ± 0.001
**C17_DHL**	185	0.07 ± 0.001	0.09 ± 0.001

### Genetic differentiation analysis

**Figure 2:**
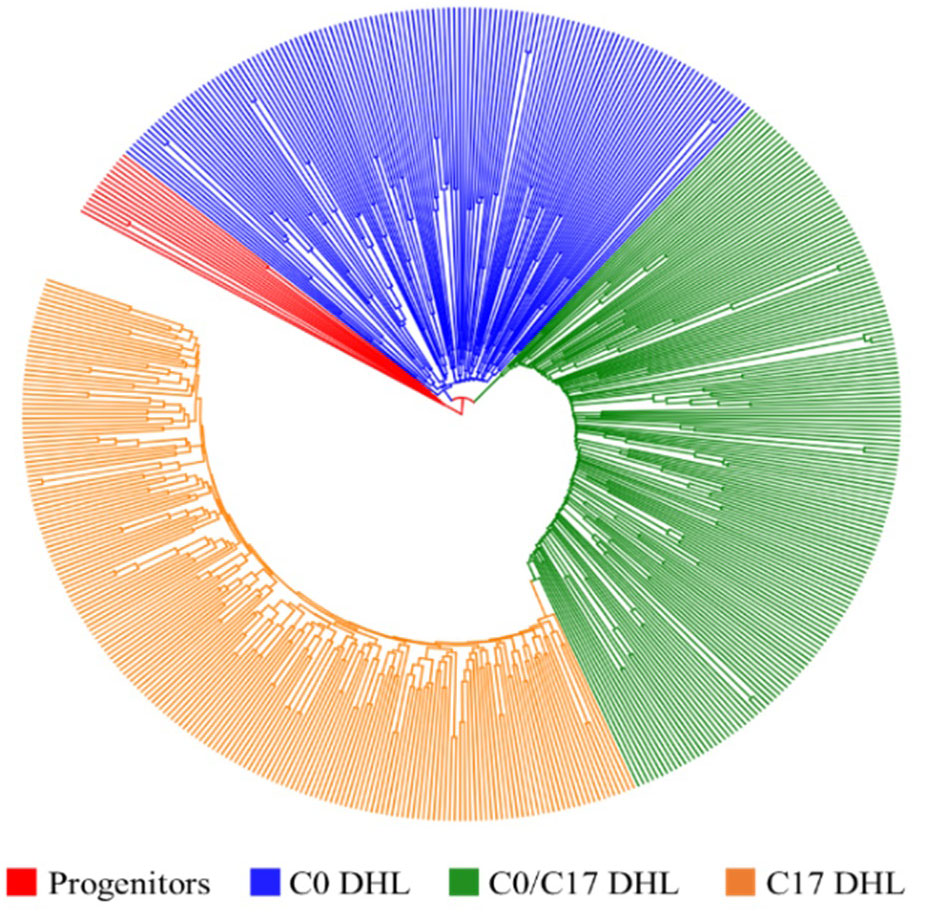
Dendrogram constructed from Euclidean genetic distance based on the UPGMA tree method for a panel of 15 progenitors and 495 DH lines derived from BSSS maize population.

**Figure 3:**
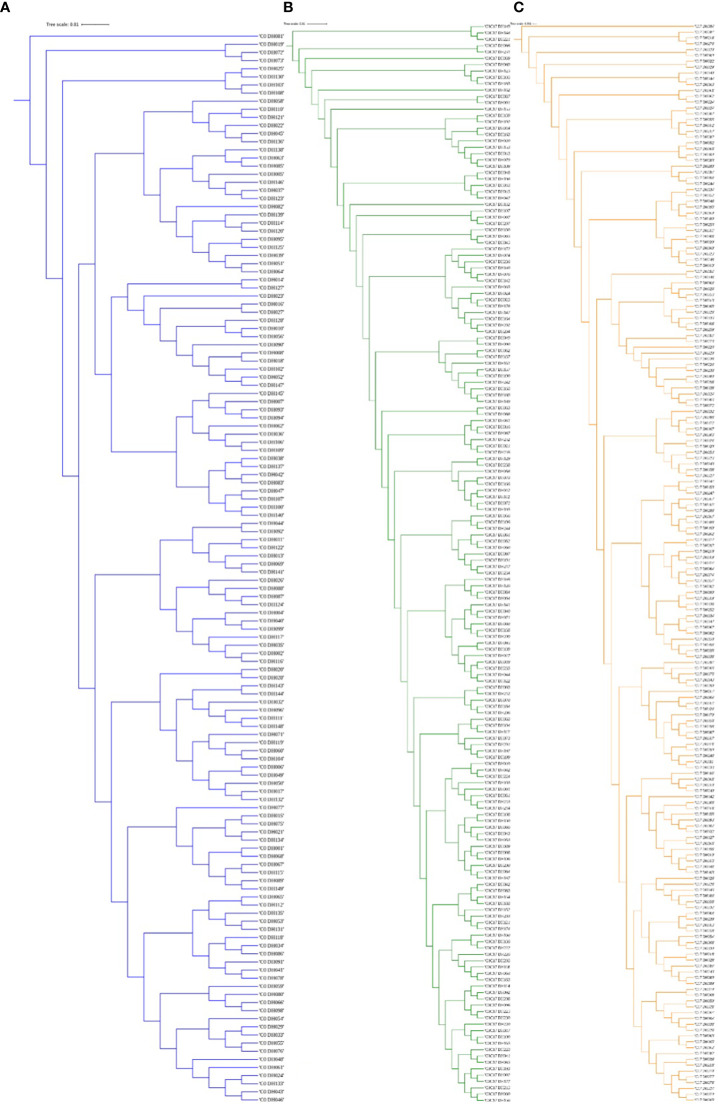
The dendrogram was constructed from Euclidean genetic distances based on the UPGMA tree method. **(A)** C0_DHL, **(B)** C0/C17_DHL and **(C)** C17_DHL of the BSSS maize population.

**Figure 4:**
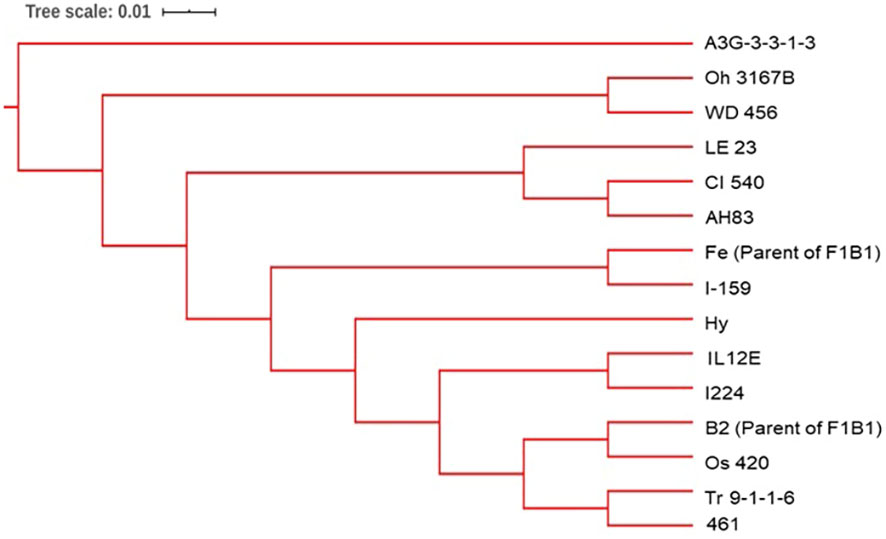
The dendrogram was constructed from Euclidean genetic distances based on the UPGMA tree method for the progenitors of the BSSS maize population.

The greatest genetic distance was observed between the progenitor group and the C17_DHL group (0.18) and the smallest genetic distance was observed between C17_DHL and C0/C17_DHL groups (0.11; **Table 3**). The UPGMA method separated the different groups of DH lines and the progenitor group (**Figures 2**–**4**). We observed that the grouping of lines and progenitors followed their origin. That is, lines and progenitors within groups were more related than among groups. In addition, we found high genetic diversity among the DH lines (C0_DHL, C0/C17_DHL and C17_DHL) and progenitors of each group.

**Table 3:** Pairwise genetic distance and degree of genetic differentiation (F_ST_) between different groups of DH lines and the progenitors of the BSSS maize population.

Group	Progenitors	C0_DHL	C0/C17_DHL	C17_DHL
**Progenitors**		0.168	0.170	0.175
**C0_DHL**	0.148		0.141	0.147
**C0/C17_DHL**	0.220	0.092		0.108
**C17_DHL**	0.496	0.340	0.131	

Below diagonal: pairwise F_ST_ estimates between different groups of DH lines and the progenitors, above diagonal: genetic distances.

**Figure 5:**
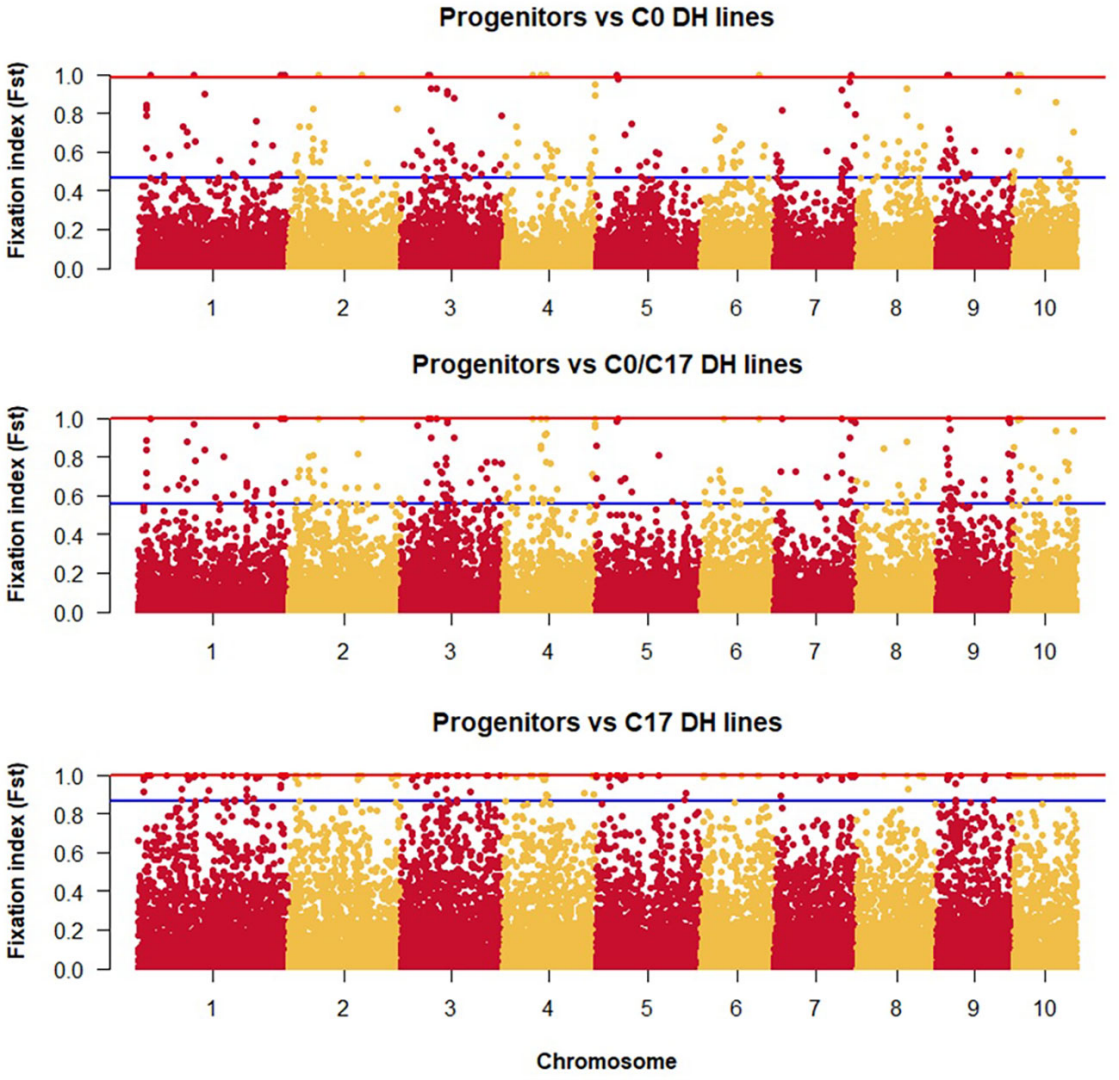
Genetic differentiation compares the progenitor group and the different groups of DH lines across chromosomes (x-axis) with the F_ST_ value (y-axis). Dots between the red and the blue lines represent the highest 1% of the F_ST_ values.

**Figure 6:**
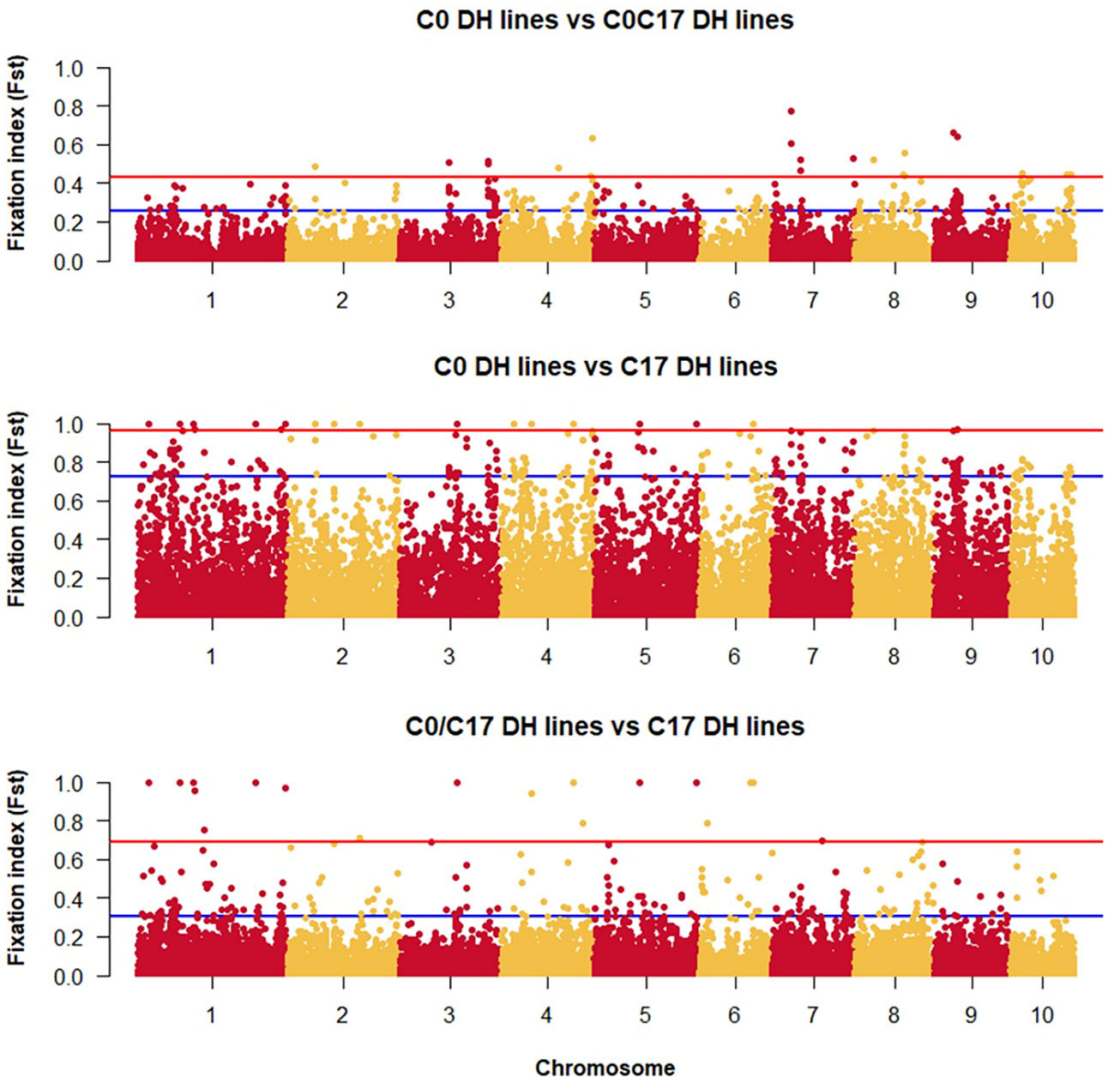
Genetic differentiation compares the different groups of DH lines across chromosomes (x-axis) with the F_ST_ value (y-axis). Dots between the rad and the blue lines represent the highest 1% of the F_ST_ values.

The lowest F_ST_ among the DH lines was observed between the progenitors and the C0_DHL group (0.15). The highest value was observed between progenitors and C17_DHL (0.50; **Table 3**). Manhattan plots showed the genetic differentiation among the different comparisons performed between the progenitors and the different groups of DH lines across the ten chromosomes, with similar patterns across chromosomes (**Figures 5** , **6**). F_ST_ values of 1 and closer to 1 were observed between the progenitor group and the C17_DHL group across the genome as expected, demonstrating a considerable differentiation.

**Figure 7:**
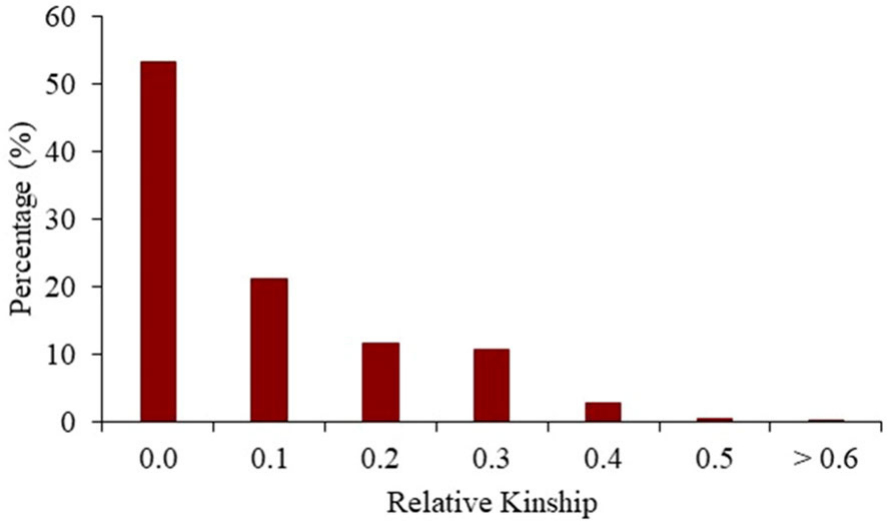
Distribution of pairwise relative kinship for 487 maize DH lines and 15 progenitors lines of the BSSS maize population calculated using 24,885 SNP markers.

In relation to the pairwise relative kinship distribution for the entire set of 487 maize DH lines and 15 progenitors, 53.2% of the kinship coefficient was equal to 0 (**Figure 7**). Whereas, 46.0% of the entire panel ranged between 0 and 0.4, and only 0.8% were greater than 0.5. Thus, most lines were either not or only distantly related to each other.

**Figure 8:**
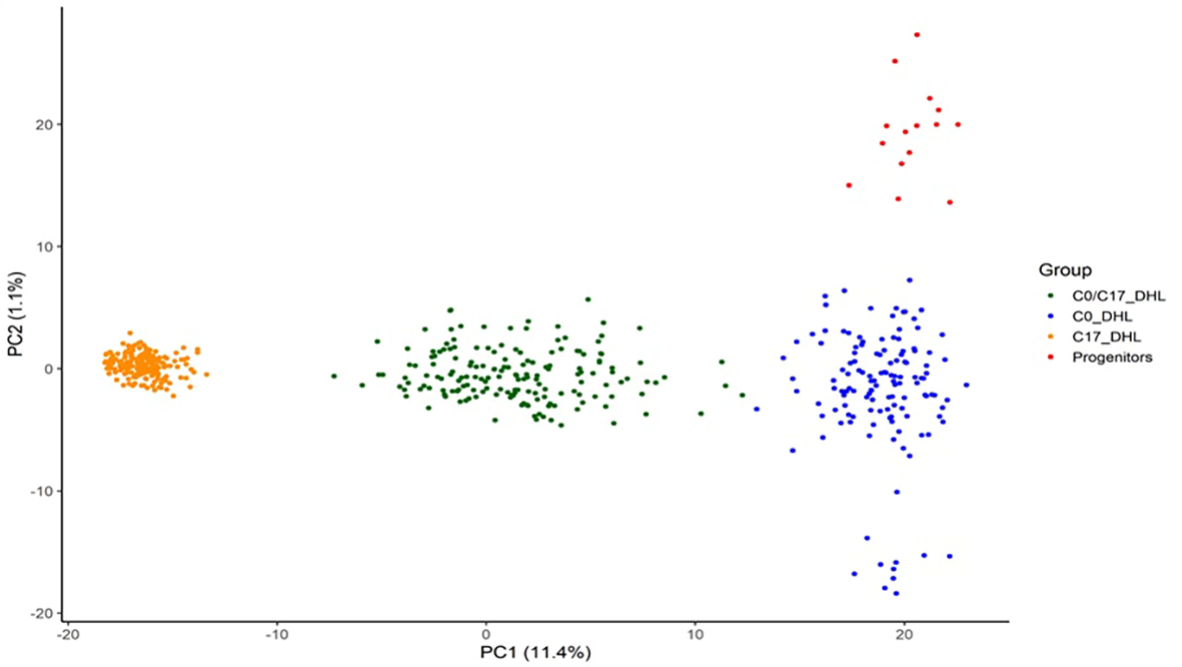
Scatter plot of the discriminant analysis of principal components based on 487 DH lines and 15 progenitors of the BSSS maize population. The dots represent each of the DH lines within their respective population. The axes represent the first two discriminant functions, respectively.

Based on PCA, DH lines developed from BSSS can be divided into three subgroups (**Figure 8**). The first two principal components explained 12.5% of the total SNP variation in the entire panel. Based on discriminant analysis of principal components (DAPC), we observed a clear grouping of the DH lines into the C0_DHL, C17_DHL andC0/C17_DHL. The progenitor lines were grouped within the C0_DHL cluster, as expected, since the combination of these 16 progenitor lines originated this population. The C0/C17_DHL group were scattered over a wider range, similar to the C0_DHL group.

**Figure 9:**
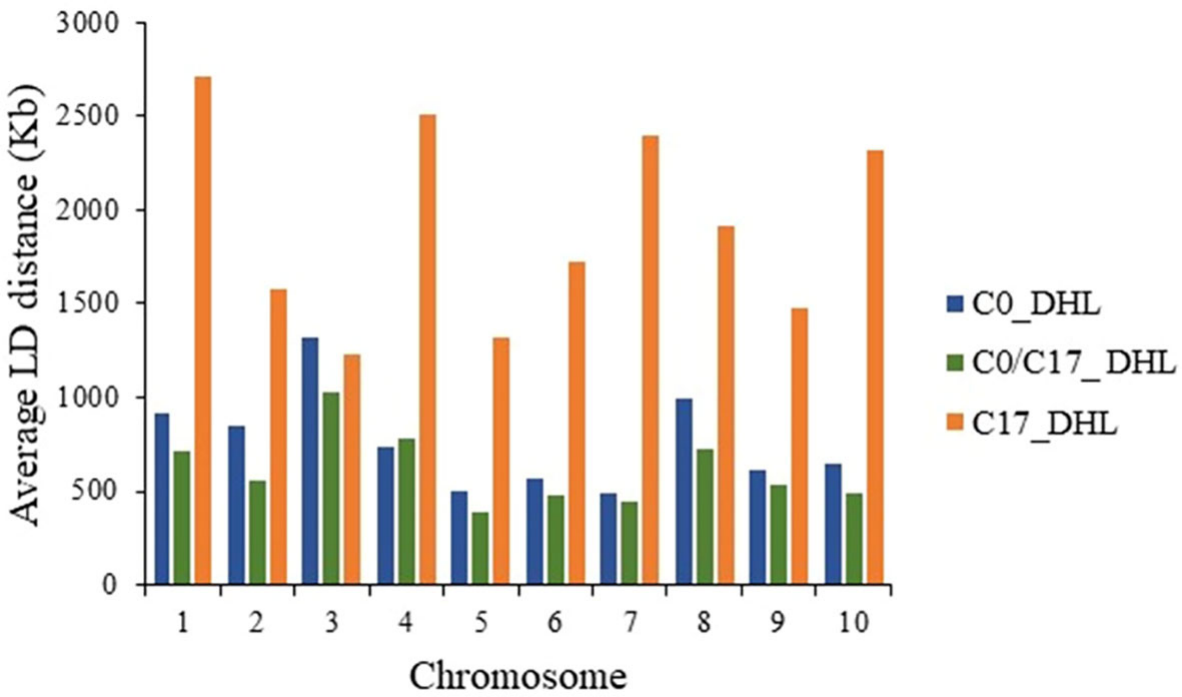
Linkage Disequilibrium (LD) decay distance per chromosome in the different groups of DH lines.

The LD decay was variable across the ten chromosomes and different genetic regions within chromosomes in each group (**Figure 9**). The C17_DHL group showed the longest LD decay distances ranging from 1,229 to 2,709 kb on chromosomes 3 and 1, respectively. In contrast, the C0/C17_DHL group displayed the shortest LD decay distances (384 kb on chromosome 5 to 1,024 kb on chromosome 3). For C0_DHL, the LD decay varied from 486 kb to 1,322 kb for chromosomes 7 and 3, respectively.

**Figure 10:**
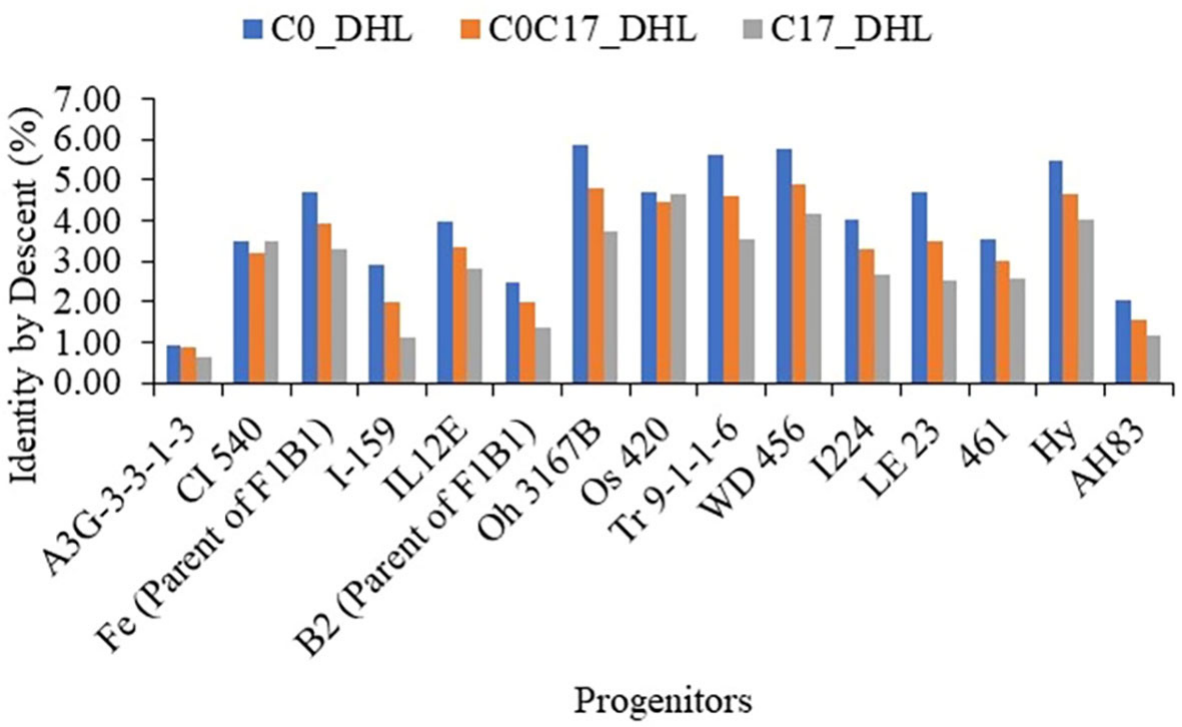
The genome’s proportion classified as IBD among the BSSS progenitors inbred lines for each group of DH lines evaluated (C0_DHL, C0/C17_DHL and C17_DHL) identified with marker-based dissimilarity values.

For the progenitors’ genetic contribution to each set of DH lines, a total of 10,344 polymorphic SNP markers distributed across the whole genome were used to estimate IBD segments among the 15 progenitors and 487 DH lines (**Supplemental Table S2**, **Figure 10**). In general, the progenitor A3G-3-3-1-3 had a low genetic contribution to the different sets of DH lines with 0.91, 0.87 and 0.63% in the C0_DHL, C0/C17_DHL and C17_DHL groups, respectively. In comparison, the progenitor WD 456 had a high genetic contribution to the different sets of DH lines with 5.76, 4.90 and 4.14% in the C0_DHL, C0/C17_DHL and C17_DH line groups, respectively. The progenitors CI 540 and Os 420 had a similar contribution to the different groups of DH lines. In general, the 15 progenitors evaluated had a higher genetic contribution to C0_DHLs, ranging from 0.91 to 5.87% for individual progenitors, compared with C0/C17_DHL (0.87 to 4.90%) and C17 (0.63 to 4.62%). The progenitor with the highest genetic contribution in C0 (Oh 3167B with 5.87%) had a lower contribution in C0/C17_DHL and C17 with 4.78 and 3.71%, respectively. On average, progenitor lines had 60.1% of the genome classified as identical by descent within C0_DHLs, 50.0% within the C0/C17_DHL and 41.6% within C17. The remaining 39.9, 50.0, and 58.4% in C0, C0/C17_DHL and C17, respectively are referred to as non-IBD markers. Those SNP markers were not included within the IBD segments between DH line groups and the progenitors.

## Discussion

Molecular markers, including SNP markers have been used in many crops including maize for characterizing and quantifying genetic diversity of a given set germplasm for further improvement in a breeding program. The analysis of genetic variation among genetic materials is important to plant breeders, as it contributes to create a core set of germplasm, selecting parental lines, assigning heterotic groups, performs association analysis and prediction potential genetic gains for traits of interest. SNP markers, due to their abundance of availability of sophisticated, rapid, and affordable high-throughput detection systems, have become the principal resource for characterizing and quantifying genetic differences within and among species.

In the present study, the final SNP marker data set included 24,885 SNPs distributed across the ten chromosomes and 502 genotypes corresponding to DH lines derived from different cycles of recurrent selection (132 C0_DHL, 185 C17_ DHL, and 170 C0/C17_DHL) plus 15 progenitors of the BSSS maize population. The rationale of using un-imputed data without filtering for MAF was that the BSSS maize population came from 16 founder genotypes. For some SNP markers, an allele was provided by only one founder. The expected frequency would in such a case be ~6.2%. If genetic drift occurred, the actual frequency in C0 can be even lower. C0 seed used in this research came from subsequent cycles of seed multiplication for maintenance, increasing the chance of genetic drift to occur.

### Changes in genetic diversity in different subsets of DH lines

When dividing the number of SNP with heterozygous loci by the total number of SNPs, we observed that our DH lines presented a very low rate of heterozygous loci (less than 3%). Therefore, our DH lines attained an appreciable level of homozygosity, and the DH technology was efficient to fix the loci without requiring further generations of purification. Higher MAF and expected heterozygosity values of the progenitor and C0_DHL groups (**Table 2**) were expected due to the large number of alleles that occurred in a few progenitor lines and were lost over recurrent selection cycles (Hagdorn et al., 2003). Additional recombination occurred because of population maintenance. Unfortunately, we do not have adequate records indicating how the seed has been maintained since 1939 when the population was created. Conversely, when comparing the C0_DHL and C17_DHL groups, we found a reduction in MAF and expected heterozygosity. The reduction in MAF among these groups was expected due to the recurrent selection process and genetic drift.

The high expected heterozygosity values found in the C0_DHL group were an indication for the presence of more rare alleles in C0. This could be an important source for new functional alleles of desirable traits, which have been lost during multiple generations of recurrent selection. Potential reduction in genetic diversity in advanced cycles were consistent with previous studies of the BSSS maize population in different cycles of the recurrent selection program (Messmer et al., 1991; Labate et al., 1997; Hagdorn et al., 2003; Hinze et al., 2005), where genome-wide genetic diversity has decreased across cycles of selection. Gerke et al. (2015) found a clear separation, when analyzing the progenitors and individuals from different cycles in the BSSS population. As this was a closed selection process, the substantial increase in genetic distance from C0_DHL to C17_DHL could only arise from genetic differentiation due to selection and genetic drift (Gerke et al., 2015).

Improvement of plant characteristics like flag leaf angle, anthesis-silking interval, plant height, tassel branch number, total number of leaves and grain yield has been observed when advancing cycles in the BSSS recurrent selection program (Brekke et al., 2011; Edwards, 2011). These changes suggest fixation of favorable alleles during the recurrent selection program. Thus, exploring BSSS cycles using DH technology may reveal useful genetic diversity for plant characteristics left behind in the recurrent selection process and could be an important resource to help drive future genetic gains in maize breeding program.

### Genetic relationship and divergence within and among cycles of selection

The Wright’s F-statistics (F_ST_) used to measure population substructure and the overall genetic divergence among the different groups showed that the degree of differentiation was higher between the progenitor inbred lines and the C17_DHL group compared to C0_DHL and C0/C17_DHL groups as expected since the two groups share fewer alleles. Lower F_ST_ values indicate limited differentiation between groups of DH lines. When we compare the F_ST_ values of C0_DHL versus C17_DHL, we observe a clear genetic differentiation among these two groups. These results can be confirmed with the wider genetic distance found among them, reflecting the uniqueness of most lines within these groups. Similar results were found by Gerke et al. (2015) when evaluating the progenitors and samples from different cycles of the BSSS maize population (C0, C4, C8, C12 and C16), indicating a clear differentiation between the founder lines and the population at C16 caused by the loss of different alleles within BSSS maize population. Gerke et al. (2015) conducted extensive simulations using BSSS founder haplotypes to gauge the roles of selection and drift among the cycles of selection and the results showed that most of the reduction in diversity observed among cycles can be attributed to genetic drift alone.

Population structure based on principal component analysis (PCA) is used to reveal genetic divergence among populations (Price et al., 2006). In this study, the results suggest a clear separation into three significant subgroups among all the BSSS DH lines and the progenitors. Also, we observed that the C0/C17_DHL group was scattered over a wide range, similar to C0_DHL, indicating a broader genetic divergence among these DH lines than for C17_DHL.

Kinship coefficients are defined by pedigree and can be estimated based on molecular information. Thus, it is possible to find hidden relationships. We found that most of the DH lines in the entire panel were distantly related to each other. Therefore, this shows us a low relationship between DH lines of the C17_DHL and C0_DHL. The estimation of the degree of the relationship depends on the description of an ancestral population, which by definition, is assumed to be the base from where the past ancestry is no longer accounted (Wright, 1922). Thus, the lower the number of generations separating the ancestral with the current population, the higher the kinship coefficient among individuals because of a reduced number of possible recombination events (Wang, 2014). Low or negative relative kinship coefficients among pairs of DH lines were found in the C0/C17_DHL group reflecting the uniqueness of most lines.

### Linkage disequilibrium in BSSS DH lines

Linkage disequilibrium (LD) refers to the non-random co-segregation of alleles at two loci. Recombination events shuffle genetic material during meiosis among homologous chromosomes and cause LD to decay with increasing distance. Multiple factors are affecting LD in crops. Generally, LD decays faster in cross-pollinated crops, diverse populations, but also, different genes and genomic regions in the same crop can exhibit different rates of LD decay. It is expected in maize, for genome regions to decay at distances around 1 kb for exotic landraces, as described by (Romay et al., 2013). In the Ames panel subset corresponding to 384 lines (Pace et al., 2015) the LD decay rate was similar across chromosomes with an average distance of 10 kb throughout the genome. In this study, the LD decay distance among lines of the C17_DHL group was larger compared among lines of the C0_DHL and C0/C17_DHL groups. The longer LD decay distances in C17_DHL was consistent with the lower average MAF and expected heterozygosity results, as the rate of effective recombination declines over selection cycles due to the occurrence of bottlenecks or due to fixation for favorable alleles over time. The 17 cycles of recurrent selection did lead to a lower genetic diversity in the C17_DHL group, and LD decays more rapidly in pools of lines with higher genetic diversity (Romay et al., 2013; Wu et al., 2016). The distance over which LD persists determines the number and density of markers, and experimental design needed to perform an association analysis (Flint-Garcia et al., 2003). This was actually applied when generating the IBM Syn10 ultra-high-density map to precisely map a quantitative trait locus (Liu et al., 2015) at a higher genetic resolution than the IBM Syn4 map (Hu et al., 2016). In contrast, additional cycles of recurrent selection in the BSSS maize population increased homozygosity and LD decay distances due to selection and drift. Consistent with Gerke et al. (2015) genome-wide expected heterozygosity decreases steadily across cycles of selection. The loss of heterozygosity indicates the loss of different alleles within BSSS maize population.

### Progenitor genetic contributions to different subsets of DH lines

On average, the mean genetic contribution of the BSSS progenitor lines estimated using high-resolution detection of IBD segments changed in the different groups of DH lines. The progenitors had the highest genetic contribution in the C0_DHL group, due to their use in obtaining the population, and the lowest contribution in the C17_DHL group in relation to the other groups. This suggests that relationships caused by more recent ancestry had the most significant contribution in the IBD segments among individuals. Additionally, 17 cycles of recurrent selection have changed the allele frequencies in the C17_DHL group, because only individuals with superior performance for the selected traits contributed alleles to the next generation.

In the identification of regions in the genome inherited from the progenitors, we found prevalence of small to medium sized segments, where 50.4% of the segments were between 2.4 to 4.1 Mb. And, 28.2% of the segments ranged from 4.1 to 8.1 Mb inherited from the progenitor inbred lines. The number of segments decreased with increased length segments. IBD segment sizes from the progenitors changed across groups of DH lines. We found that some progenitors showed longer IBD segments in the C0_DHL group and others longer in the C17_DHL. Large, preserved regions in the genome could be associated with selection processes, resulting in long DNA segments inherited as a block from the parents. Therefore, under positive selection favoring a phenotype, a slight increase in LD surrounding the favored alleles will be produced. In these cases, the length of the IBD segment surrounding the alleles subject to selection will increase, experiencing less recombination at the population level (Albrechtsen et al., 2010). Albrechtsen et al. (2010) states that a reduced recombination rate in the genome, leading to significant LD, could be explained as a function of the effective population size. These could partially be explained by an increase in random genetic drift because of the population size, which will increase the length of DNA that will be shared among individuals in the population similar to what could happen in the C17_DHL with the 17 cycles of the recurrent selection process. The detection of long IBD segments in populations could be used as evidence for strong and recent selection processes because these segments have not suffered from recombination. However, many recombination’s could have occurred because of subsequent cycles of seed multiplication and population maintenance. Unfortunately, we do not have adequate records indicating how the seed has been maintained since 1939 when the population was created. In cases where alleles within long IBD segments are in linkage disequilibrium, specifically in repulsion phase, unfavorable alleles will persist in the population, inducing the hitch-hiking effect and reducing the genetic diversity (Hospital and Chevalet, 1993). This hitch-hiking will increase genetic drift and significantly decrease the effective population size (Smith and Haigh, 1974). More studies should necessarily be done to confirm the possibility of the hitch-hiking effect having an effect in this population. Conversely, the restricted population size of both from founding (16 lines) and from continued population maintenance, may have provided the maintenance of long IBD segments. IBD segments shared between different groups of DH lines and the 15 progenitor lines will allow the estimation of genetic diversity and progenitor genetic contributions to new released lines.

In this study, we measured the genetic diversity among different sets of DH lines derived from the BSSS maize population and our results confirmed the separation from BSSS(R)C0 to BSSS(R)17 through the recurrent selection process. The selection process and the effective population size applied to the BSSS maize population have reduced the genetic variability. Consistent with previous studies (Messmer et al., 1991; Labate et al., 1997; Hagdorn et al., 2003; Hinze et al., 2005). Although genetic drift can explain most of the genetic structure genome-wide, phenotypic data provide evidence that selection has altered favorable allele frequencies in the BSSS maize population. We also found that the greatest genetic distance and F_ST_ observed between the progenitors group and the C17_DHL group demonstrated a clear genetic differentiation among groups caused by the loss of different alleles during the recurrent selection program in the BSSS maize population, reflecting the uniqueness of most lines within these groups of DH lines. Thus, these DH lines can be evaluated in replicated trials, and genomic selection can be applied for the estimation of the breeding value for each DH line. Additionally, DH lines derived from the BSSS maize population could be ideal for association mapping due to the low population structure. Thus, we could identify genes or regions in the genome associated with a particular trait. Using genome-based data and DH technology was a powerful tool for access to the genetic diversity available in C0_DHL or C0/C17_DHL groups, which would be beneficial to incorporate in BSSS(R)17 to broaden its genetic variation while minimizing yield or other penalties. Thus, the results of this research will also help maize breeders to explore useful genetic variation for further improvement in a breeding program.

## Data availability statement

The datasets presented in this study can be found in online repositories. The names of the repository/repositories and accession number(s) can be found below: Iowa State University DataShare, accession 22893878. DOI: https://doi.org/10.25380/iastate.22893878.v1.

## Author contributions

TL, AL, JE, UF and SH conceived and designed the experiments. AL analyzed the genotypic data and conducted the molecular characterization. AL and FA conducted the IBD analysis. AL, AU and TL wrote the manuscript, with contributions from all the other authors. All authors contributed to the article and submitted and approved the submitted section.
